# The Impact of the Green Credit Policy on the Short-Term and Long-Term Debt Financing of Heavily Polluting Enterprises: Based on PSM-DID Method

**DOI:** 10.3390/ijerph191811287

**Published:** 2022-09-08

**Authors:** Yan Yang, Yingli Zhang

**Affiliations:** School of Economics and Management, Shanghai Ocean University, Shanghai 201306, China

**Keywords:** Green Credit Policy, heavily polluting enterprises, debt financing, difference-in-difference method, propensity score matching, China

## Abstract

“Green economy and sustainable development” has become the focus of contemporary world economic development. As an important part of green financial instruments, green credit has become a hot topic. This paper investigates whether the Green Credit Policy has had any impact. Does it have a binding effect on the debt financing of heavily polluting enterprises? Using the *Green Credit Guidelines* as the starting point for the implementation of the Green Credit Policy, this paper takes Chinese A-share listed enterprises from 2004 to 2020 as the research sample, and applies the propensity score matching combined with difference-in-difference (PSM-DID) method to analyze the impact of green credit policies on the long- and short-term financing scale of heavily polluting enterprises. The study found that the implementation of the Green Credit Policy significantly suppressed the long-term financing of heavily polluting enterprises, but allowed for the expansion of short-term financing for heavily polluting enterprises. Compared with the state-owned enterprises, the Green Credit Policy has a more significant impact on non-state-owned enterprises in terms of suppressing long-term financing and increasing short-term financing, suggesting that the Green Credit Policy is affected by the “credit discrimination” of non-state-owned enterprises. Therefore, the Green Credit Policy still needs to be improved. This study provides empirical evidence of the effectiveness of green credit policies in China, and offers suggestions for further green credit policies in the future.

## 1. Introduction

China’s economy has grown rapidly since its reform and opening-up, but environmental pollution and energy scarcity are becoming increasingly serious. In the early 1990s, less than 1% of the more than 500 cities in China met the National Air Quality Standard Level 1; in the early twenty-first century, nearly two-thirds of the urban population was exposed to poor air quality [1]. According to the ninth National Forest Inventory, China’s current forest coverage rate is 22.96%, far below the global average of 30.6%, and the per capita forest area is 0.16 hm^2^, less than one-third of the global level [2]. From a global perspective, China is a country with low per capita ecological wealth, but its energy consumption and carbon dioxide emissions have surpassed those of the United States to become the world’s largest, resulting not only in a massive negative externality to the world, but also in a serious separation from China’s scarcity of per capita resources, and a fragile ecological environment within the nation [3].

Faced with the enormous challenge of resource and environmental problems, China has begun to attach importance to the construction of ecological civilization, insisting on harmonious coexistence between humans and nature, and establishing and practicing the idea that green water and green mountains are ideals to be aimed for, adhering to the basic state policy of saving resources and protecting the environment, and formally aspiring to carbon peaking and carbon neutrality in 2020 [4]. As a proponent of the “community of human destiny”, China has enacted a series of green development policies, including the Green Credit Policy.

The concept of “green credit” was first introduced in 2007 in the *Opinions on Implementing Environmental Protection Policies and Regulations to Prevent Credit Risks*, and in 2012 the China Banking Regulatory Commission (CBRC) issued the *Green Credit Guidelines*, marking the beginning of a comprehensive development phase for green credit in China, gradually moving from the theoretical to the practical level [5]. This was followed by the *Introduction of Green Credit policies such as key indicators for the implementation of Green Credit* and the *Guidance on the construction of a green financial system* [6]. Today, China ranks first in the world in terms of the scale of green credit.

Green credit refers to the use of interest rates leverage by banking financial institutions to control the flow of credit funds based on complying with industrial policies, by limiting the productive and investment activities of enterprises that consume a lot of energy and emit a lot of pollution, to guide financial resources to gradually flow towards low-carbon, clean enterprises, and thus achieve the “green allocation” of funds via carbon emissions control [7,8]. Its primary function is to encourage the green transformation and upgrading of businesses and industries, as well as to promote China’s green economic growth.

The Chinese government advocated for “resolutely restraining the blind expansion of energy-intensive and high-emission projects” and “vigorously promoting Green Finance” in its 14th five-year plan [9]. This leads to the question, has a Green Credit Policy been established since its inception? In this context, this paper attempts to assess the effect of the Green Credit Policy from the perspective of long-term and short-term financing. It is of great theoretical and practical significance to study the impacts of green credit on the financing constraints of enterprises.

The research contribution of this paper is mainly reflected in the following three aspects:

Firstly, in the context of China’s current push for green economic growth, the existing literature on the Green Credit Policy mainly approaches the issue from the macro level and bank perspective [10,11], while the research based on the enterprise micro-perspective mainly focuses on enterprise green innovation [12,13,14], but the research on the impact of the Green Credit Policy on enterprise financing falls short. This paper introduces short-term debt financing and long-term debt financing to examine the financing constraints of the Green Credit Policy on heavily polluting enterprises, which is conducive to evaluating the effectiveness of the implementation of the Green Credit Policy from a more diversified perspective.

Secondly, while most scholars currently use difference-in-difference methods directly in their policy effect studies [15,16,17], this paper applies propensity score matching to process the data, and selects a new treatment group and control group with similar covariates from the original sample, making the distribution of the two groups balanced.

Thirdly, this paper explores the impact of the Green Credit Policy on the debt financing relationships of heavily polluting enterprises with different ownership properties, reveals the current situation of credit discrimination, and provides recommendations in the Discussion section in response to the findings of the study, which have important practical implications for the sustainable development of the Green Credit Policy.

The rest of this paper is organized as follows: the second part is a literature review; the third part is a hypothesis proposal; the fourth part is the model specification; the fifth part is the results and discussion; the sixth part is the discussion; and the final part is the research conclusion.

## 2. Literature Review

To test the effectiveness of green credit policies, scholars have conducted a number of studies on green credit at the macro and micro levels. At the macro level, the discussions of the effects of the Green Credit Policy implementation have mainly focused on pollutant emissions and green economic growth. Guo et al. found that green credit helped to enhance the effectiveness of China’s green economy, and positively contributed to regional economic growth [18,19,20], and this promotion also had spatial spillover effects [21]. The implementation of green credit policies helped to reduce pollutant emissions at both the national level and at the level of heavily polluting industries [22,23], reducing carbon and sulfur dioxide emissions and curbing PM2.5 concentrations [24,25,26].

At the micro level, the research mainly approaches from the bank and enterprise perspective. At the bank level, green credit has effectively improved the financial performance of banks [27,28], with some scholars finding that the efficiency of green credit as employed by banks still needs to be improved [29], while others have found that state-owned banks actually sacrifice their profitability to provide green credit, and that risk management should not prevent banks from granting green credit, reflecting China’s commitment to green credit policies [30]. At the enterprise level, most studies have focused on green innovation, and green credit policies have enhanced green innovation overall [31,32]. There are many studies focusing on heavily polluting enterprises, but the results are divided into two schools of thought, with some studies showing that green credit has promoted innovation in heavily polluting enterprises [33,34,35], and others finding that green credit policies have hindered green innovation in heavily polluting enterprises [36,37,38]. Green credit policies have promoted investment in environmental management and corporate social responsibility among heavily polluting enterprises [39,40]. However, the current research on the impact of green credit on corporate finance is incomplete and lacks validation.

The financing behavior of enterprises is very important to the development of enterprises. Earlier studies have found that green credit policies have a limited impact on the cost of financing high-polluting, high-emission firms; the main reason is that most of the high-pollution, high-emission industries are enterprises with heavy assets, short-term profits and good stability, or are local pillar enterprises [41]. With the gradual improvement of the Green Credit Policy, some scholars have found that the policy has increased the debt financing costs of high-polluting and high-emission enterprises in recent years [42,43], and some scholars have also studied the background of the Green Credit Policy, green management, the quality of accounting information and the relationship between enterprise financing. They found that heavy-pollution enterprises did not provide a higher quality of accounting information to obtain lower bank loan costs, and this shows that the Green Credit Policy has some effect [44]. Liu et al. found that the Green Credit Policy has reduced the total amount of financing available to heavily polluting companies [45]. At present, scholars mainly use financing costs to measure the impact of the Green Credit Policy on enterprise financing, and some scholars also use the scale of financing for this purpose, but the impacts of green credit policies on short-term and long-term corporate finance remain to be elucidated. The existing empirical literature is rarely discussed, and is the focus of this paper.

Based on this, this paper uses the PSM-DID method to investigate whether the implementation of the Green Credit Policy significantly reduces the long-term and short-term debt financing of heavily polluting enterprises, and whether they have financing constraints. This paper can reflect the real short-term and long-term financing levels of enterprises to a considerable extent, and can provide a new perspective and empirical evidence for studying the effects of green credit policies, enriching the existing relevant literature and providing empirical evidence useful for bank credit decisions and regulatory authorities.

## 3. Hypothesis Proposed

When entrepreneurs have sufficient information about their businesses, “internal financing debt financing as equity financing” is regarded as the optimum financing sequence, according to Modigliani and Miller [46]. When an enterprise’s internal capital is insufficient to maintain its development, it must seek external financing. Short-term and long-term debt financing are two types of debt financing.

Short-term financing is typically employed for temporary purposes, has strong liquidity, and is referred to as “self-liquidating”. Furthermore, the asset structure and operating conditions of the enterprise will not change greatly in the short-term, which helps the creditor maintain control over the debtor’s repayment risk, and the short-term financing risk is reduced [47]. Shortening the financing time, according to agency cost theory, can help to limit managers’ abuse of cash flow, encourage managers to pay more attention to fund efficiency, and help to optimize the governance structure of polluting enterprises [48]. According to information asymmetry theory, low-risk, high-quality enterprises will first choose short-term financing, gradually reducing the information asymmetry and increasing the financing term by sending out relevant signals, whereas some high-risk polluting enterprises will prefer long-term financing to avoid the extra costs of short-term financing. At the same time, there is a positive relationship between a firm’s size and the duration of its financing period [49], meaning that under the Green Credit Policy, banks are required to restrict long-term financing in order to reduce their own risk and control the expansion of polluting firms, whilst expanding short-term financing. Low-credit enterprises, according to Diamond’s [50] model, are unable to secure long-term financing due to adverse selection factors, hence they must rely on short-term financing. Banks are more motivated to issue short-term loans to retain their prestige by not renewing loans, which is advantageous to the restriction and supervision of polluting enterprises.

According to the Green Credit Policy, environmental risk must be factored into enterprise credit rating standards. If an enterprise fails to pass the environmental assessment and approval process or the acceptance check performed by environmental protection facilities because it does not conform to the industrial policy or has committed environmental violations, there are immediate environmental risks. First, according to the theory of environmental risk, environmental pollution in the course of business activities leads to environmental deterioration and resource consumption, which contradicts the concept of sustainable development. Second, heavy-pollution enterprises must pay fines for environmental violations, or face losses resulting from rectification and reconstruction, which has a significant impact on cash flow and raises the risk of debt default. Third, if the scale of borrowing is not restricted, it will pose a reputation risk in the context of the Green Credit Policy. Therefore, to avoid risk, the bank may significantly reduce the long-term financing of heavily polluting enterprises, whereas short-term financing can reduce the bank’s risks, meaning that the overall impact is unclear. The following hypotheses are proposed based on the above analysis:

**Hypothesis** **1.** *The Green Credit Policy offers an incentive towards short-term financing for heavily polluting enterprises*.

**Hypothesis** **2.** *The Green Credit Policy poses a significant disincentive to long-term financing for heavily polluting enterprises*.

In terms of bank loan financing, Chinese state-owned enterprises tend to have easier access to bank credit policy [51]; often, all it takes is proof that the business is financially sound, and that the project is viable, to gain credit approval [52]. At the same time, private enterprises are inevitably subject to property rights discrimination [53]. One reason is that banks are reluctant to allocate funds to SMEs due to risk considerations. The other reason is that private SMEs are less subjected to the requirements of financial management and commercial reputation in the process of development, and can more easily meet the bank’s guarantee requirements and the regulatory requirements of relevant institutions [54].

Information asymmetry conditions exist in the credit market, and banks are prone to moral hazards and adverse selection. When banks provide credit, they tend to choose less risky state-owned enterprises over higher-yielding non-state-owned enterprises.

Because state-owned enterprises bear a great deal of responsibility for addressing employment and social stability issues, the government will provide corresponding financing support to state-owned enterprises, even if a bankruptcy crisis occurs as a result of poor management, to alleviate their debt problems. However, private enterprises are not treated the same as state-owned enterprises in the credit market, and credit discrimination occurs. The role of the Green Credit Policy may be especially crucial for non-state-owned enterprises, which are vulnerable to credit fund disbursement. The following hypotheses are proposed based on the preceding analysis:

**Hypothesis** **3.** *The Green Credit Policy has a greater impact on short-term financing for non-state-owned enterprises than for state-owned enterprises*.

**Hypothesis** **4.** *The Green Credit Policy has a more significant disincentive effect on long-term financing for non-state-owned enterprises than for state-owned enterprises*.

## 4. Model Specification

The research sample used in this study includes enterprises listed on China’s A-share market from 2004 to 2020.

This paper compiles the data from CSMAR (China Stock Market and Accounting Research Database) and uses the panel data of A-share listed enterprises from 2004 to 2020 as the research sample, excluding financial class listed companies, ST class listed companies, and some samples missing serious financial data. The treated group comprised heavily polluting listed enterprises, while the control group comprised non-polluting enterprises. Finally, a total of 8797 observations was gathered. The treatment group contained 3942 observations and the control group contained 4855 observations. Excel 2016 (Microsoft Corporation, Shanghai, China) and STATA16.0 software (Stata Corporation, Beijing, China) were used to process the data.

### 4.1. Variable Selection

1. Explained variables. This article selects short-term loans and long-term loans as the variables to be interpreted, wherein short-term financing is the explanatory variable for Hypotheses 1 and 3, and long-term financing is the explanatory variable for Hypotheses 2 and 4.
2. Explanatory variables. In 2012, the former CBRC issued the *Green Credit Guidelines*, which marked the beginning of the Green Credit Policy, meaning that the time virtual variable will be 1 in 2012 and later, and 0 before 2012.

In conjunction with the *Management List of Environmental Protection Verification Industries for Listed Companies* issued by the General Office of the Ministry of Environmental Protection in 2008, the sectoral classification of the *National Economic Industry Classification GB/T 4754-2017*, and the *Guideline on Industry Classification of Listed Companies* revised by the SFC in 2012, the following industries are classified as heavy polluting industries in this paper: mining (B), wine, beverage and refined tea manufacturing (C15), tobacco (C16), the textile industry (C17), leather, fur, feather and feather products and footwear industries (C19), the paper and paper products industry (C22), the petroleum, coal and other fuel processing industries (C25), the chemical raw materials and chemical products manufacturing industry (C26), the pharmaceutical manufacturing industry (C27), the chemical fiber manufacturing industry (C28), the rubber and plastic products industry (C29), the non-metallic mineral products industry (C30), the ferrous metal smelting and rolling processing industry (C31), the non-ferrous metal smelting and rolling processing industry (C32), and the electricity, heat, gas and water production and supply industry (D); listed companies within the above industries have been taken as 1, and the rest as 0.

3. Control variables. A set of control variables that have been shown to affect corporate debt financing has been selected to measure the impact of the Green Credit Policy and to test the hypothesis proposed in this paper. It includes the basic indicators of corporate age [55] and corporate size [56], profitability [53] using the yield valve and liquidity ratio [57], the debt service capacity using the asset liability ratio and the cash flow interest protection multiple [58], and so on.

### 4.2. Model Specification

Difference-in-difference (DID) can solve the bias in policy effect evaluation caused by other factors, and avoid the endogenous problem of policy as the explanatory variable. Therefore, this paper used DID to study the effect of the Green Credit Policy on the financing of heavily polluting enterprises. Based on the availability of data, the samples are all listed companies. The samples are divided into the treatment group and the control group, the treatment group for heavily polluting enterprises and the control group for non-heavily polluting enterprises. Since the division between the treatment group and the control group was not randomly selected, to avoid the presence of different characteristics between the treatment group and the control group, the method of propensity score matching combined with difference-in-difference (PSM-DID) proposed by Heckman et al. [59] can allow the samples to satisfy the common trend hypothesis. Using 2012 as the cut-off point to distinguish the two stages before and after the implementation of the policy, the DID model is constructed:

(1) $$Y_{\mathrm{it}}=\beta_{0}+\delta_{1}{\mathrm{Treat}}_{i}+\delta_{2}{\mathrm{Period}}_{t}+\beta_{1}{\mathrm{Treat}}_{i}\times {\mathrm{Period}}_{t}+{\alpha \mathrm{Controls}}_{\mathrm{it}}+\varepsilon_{\mathrm{it}}$$

Taking short-term financing and long-term financing as the explained variables ($Y_{\mathrm{it}}$), the subscript i and the subscript t represent the security code and the year, respectively; the virtual variable ${\mathrm{Treat}}_{i}$ reflects whether the enterprise is a heavy polluter, and the value of 1 represents heavy-polluting enterprises, while the value of 0 represents non-heavily polluting enterprises; the virtual variable ${\mathrm{Period}}_{t}$ reflects whether the enterprise is affected by the Green Credit Policy and ${\mathrm{Period}}_{t}=1$ represents the year after the implementation of the Green Credit Policy, that is, 2012 to 2020, while ${\mathrm{Period}}_{t}$ = 0 represents those not affected by the Green Credit Policy, that is, before 2012. The interaction term is ${\mathrm{Treat}}_{i}\times {\mathrm{Period}}_{t}$, in which the value of heavily polluting enterprises is 1 after the implementation of the Green Credit Policy and 0 before; ${\mathrm{Controls}}_{\mathrm{it}}$ are other control variables, including the age of the enterprise, the total assets of the enterprise (taken as logarithm), the return on net assets, the current ratio, the cash flow ratio, the asset–liability ratio, and the cash flow interest guarantee multiple. $\varepsilon_{\mathrm{it}}$ stands for random interference. The specific meanings of each variable are shown in Table 1.

Table 2 shows the parameters of the DID model. The range of change in the treatment group before and after the policy is $\delta_{2}+\beta_{1}$, and the range of change in the control group after the policy is $\delta_{2}$. For the above Model (1), this paper focuses on the estimates of $\beta_{1}$, measuring the impact of the Green Credit Policy on the short-term and long-term financing of listed companies. If the implementation of the Green Credit Policy restrains the short-term and long-term financing of heavily polluting enterprises, then the coefficient of $\beta_{1}$ is significantly positive, and the opposite means it is significantly negative.

To further study whether the property of enterprise ownership will affect the implementation effect of the Green Credit Policy, that is, to explore whether Hypotheses 3 and 4 are valid, the virtual variable Ownership is introduced on the basis of the above-mentioned DID model (1), where Ownership=1 represents the state-owned enterprise, and Ownership=0 represents non-state-owned enterprises and is modeled as:

(2) $$Y_{\mathrm{it}}=\beta_{0}+\delta_{1}{\mathrm{Treat}}_{i}+\delta_{2}{\mathrm{Period}}_{t}+\beta_{1}{\mathrm{Treat}}_{i}\times {\mathrm{Period}}_{t}\times \mathrm{Ownership}+{\alpha \mathrm{Controls}}_{\mathrm{it}}+\varepsilon_{\mathrm{it}}$$

## 5. Results and Discussion

### 5.1. Propensity Score Matching Results

In this paper, the PSM method is used to match the treatment group and the control group. Age, C_ratio, CC_ratio, CF_ratio, Lev, roe, growth, and size are selected as the matching variables to solve the problem of sample selection; thus, the assumption of the common trend of DID is satisfied.

**Figure 1:**
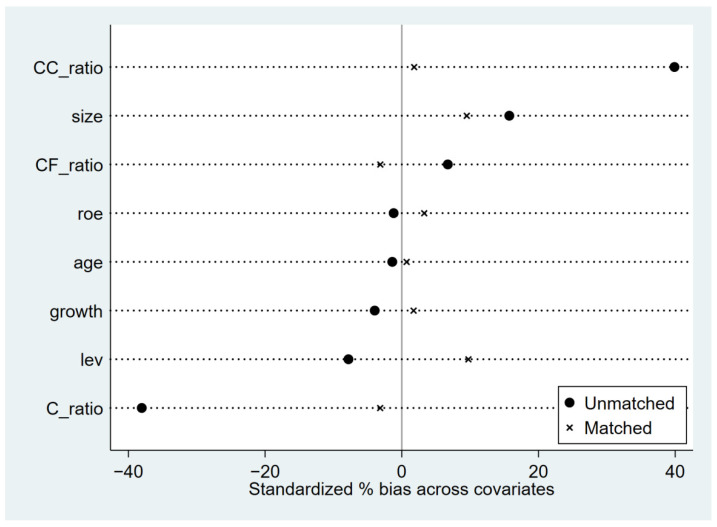
Comparison of standard deviation before and after matching.

The stability of the PSM method is mainly tested by observing the standard deviation and t-statistics. According to Figure 1, it can be seen that the standard deviations after matching become significantly smaller, and are distributed around 0. From the matching effect test shown in Table 3, it can be seen that the standardized deviation corresponding to all covariates after matching is less than 10, close to 0, and the deviation between variables is reduced. In addition, the results of the *t*-test show that C_ratio, CC_ratio, CF_ratio, and Growth were significantly different between the treatment and control groups before matching, and no longer significantly different after propensity score matching was performed. The results show that the characteristics of the treatment group and the control group were very similar after kernel matching, and propensity score matching balanced the data.

**Figure 2:**
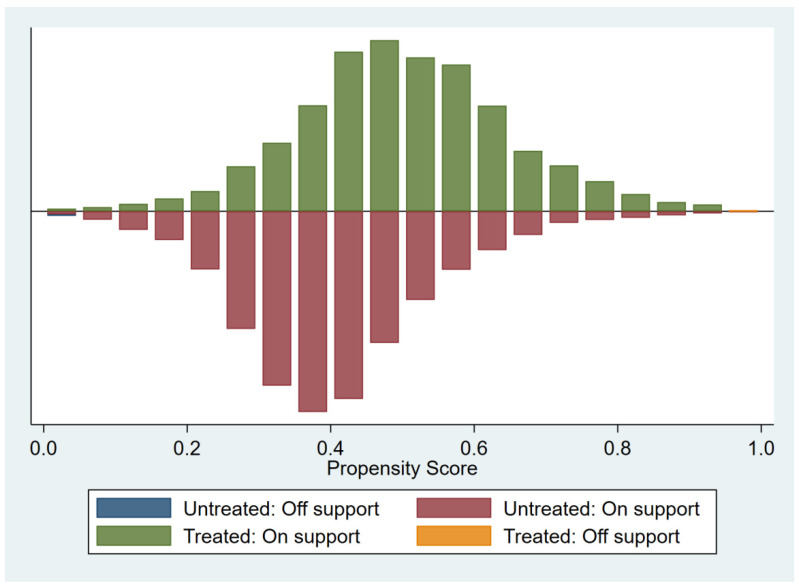
Distribution of the PSM results.

As can be seen from Figure 2, the comparison between the treatment group and the control group yields a large common range. It is believed that after calculating the degree of propensity score match, the individuals in the treatment and control groups will find corresponding matched objects, and almost no data will be lost.

The descriptive statistical results for each variable are reported in Table 4. The short-term loan and long-term loan have means of 0.174 and 0.0844. The maximum values are 0.534 and 0.465, respectively, the minimum values are both 0, and the standard deviations are 0.111 and 0.102, respectively. This demonstrates that the difference between short-term financing and long-term financing is minor.

### 5.2. Analysis of DID Model

#### 5.2.1. Regression Results and Analysis of the Effects of the Green Credit Policy on the Short-Term and Long-Term Financing of Heavily Polluting Enterprises

The PSM method was used to create a new treatment group and a control group, which were then analyzed using the DID method. Table 5 shows the DID model test results, where DID represents the treatment effects of the Green Credit Policy on heavily polluting enterprises, with columns (1) and (3) being estimates that do not include other control variables, and columns (2) and (4) are the results including other control variables. The empirical analysis results show that the coefficient of the interaction term DID is significant regardless of whether other control variables are included.

Columns (1) and (2) in Table 5 report the impact of the implementation of the Green Credit Policy on the short-term financing of heavily polluting enterprises, without the addition of other control variables, and the effect of the Green Credit Policy is significantly positive on the short-term financing of heavily polluting enterprises at the 1% level; after controlling for variables, the R-squared is significantly increased from 0.038 to 0.271, which means that the control variable enables the model to achieve a better fitting effect. The interaction term coefficient is 0.022 and is significant at the level of 1%. The results show that the implementation of the Green Credit Policy causes the short-term financing of heavily polluting enterprises to increase significantly, meaning that Hypothesis 1 holds.

Columns (3) and (4) in Table 5 report the impact of the implementation of the Green Credit Policy on the long-term financing of heavily polluting enterprises without controlling for the relevant variables, and show that the effect of the Green Credit Policy is significantly negative on the long-term financing of heavily polluting enterprises at the 1% level; after controlling for the relevant variables, the R-squared is significantly increased, the coefficient of the interaction term is −0.023, and it is significant at the 1% level. It can be concluded that the implementation of the Green Credit Policy has a restraining effect on the long-term financing of heavily polluting enterprises whether or not control variables are included. In conclusion, Hypothesis 2 is verified.

The regression coefficients of the control variable, Lev, in columns (2) and (4) are 0.156 and 0.207, which are significant at the level of 1%, indicating that the higher the asset–liability ratio, the greater the short-term and long-term financing. In column (2), all the control variables except Age are significant at the 1% confidence level, which shows that the choice of Model 1’s control variables is reasonable.

#### 5.2.2. The Effect of Ownership on the Relationship between the Green Credit Policy and the Short-Term and Long-Term Financing of Heavily Polluting Enterprises

To further study whether the nature of enterprise ownership can regulate the financing of enterprises under the Green Credit Policy, the enterprises in the PSM sample are divided into state-owned enterprises and non-state-owned enterprises; state-owned enterprises contained 5408 observations, while non-state-owned enterprises contained 3389 observations.

Column (1) and column (2) in Table 6 show that the interaction term coefficient between state-owned enterprises and non-state-owned enterprises is significant at the level of 1%, 0.022 for state-owned enterprises, and 0.026 for non-state-owned enterprises. Compared with state-owned enterprises, the effect of the Green Credit Policy on the expansion of the short-term financing of non-state-owned enterprises in heavily polluting enterprises is obvious, meaning that Hypothesis 3 is verified.

Column (3) and column (4) show that the interaction term coefficient is significantly negative at the level of 1%. The effect of the policy on state-owned enterprises is −0.021, and the effect on non-state-owned enterprises is −0.026. Therefore, compared with the state-owned heavily polluting enterprises, the policy has a more significant inhibiting effect on the long-term financing of non-state-owned enterprises. The empirical results agree with the expected judgment of Hypothesis 4.

The regression coefficients of the control variable, Lev, are significantly positive at the 1% level, indicating that the higher the asset–liability ratio, the greater the short-term and long-term financing. Most of the control variables in Table 6 are significant at the 1% confidence level, which shows that the selection of Model 2’s control variables is reasonable.

### 5.3. Robustness Test

#### 5.3.1. Propensity Score Matches

In the former paper, kernel matching was used in propensity score matching. Here, the samples are matched again via 1:4 nearest neighbor matching and caliper matching, and then DID regression is carried out. According to the regression results in Table 7, we can see that the interaction term coefficient is still significant at the 1% level, and the Green Credit Policy can restrain long-term financing and promote short-term financing. There is no substantial difference between the results of regression using the kernel matching method and those of the previous method, which shows that the experimental results are robust.

#### 5.3.2. Placebo Test

To further verify that the empirical results were not caused by chance events, a placebo test is conducted. In this paper, based on Model (1), the treated group is randomly selected for the experimental test and the random sample is repeated 500 times. The estimated value of the DID coefficient $\hat{\beta_{1}}$ is expressed as follows:

(3) $$\hat{\beta_{1}}=\beta_{1}+\gamma \frac{\mathrm{cov}({\mathrm{DID}}_{\mathrm{it}},\varepsilon_{\mathrm{it}}|{\mathrm{control}}_{\mathrm{it}})}{\mathrm{var}({\mathrm{DID}}_{\mathrm{it}}|{\mathrm{control}}_{\mathrm{it}})}$$

${\mathrm{control}}_{\mathrm{it}}$ represents all the control variables involved above, and γ represents the effect of non-observable factors. If $\hat{\beta_{1}}$ is unbiased, then γ=0. However, as it is not possible to directly estimate whether γ is 0, this paper uses a computer simulation of randomly selected experimental groups to verify whether DID has an effect on the short-term and long-term debt financing of heavily polluting enterprises. Via this method, if $\hat{\beta_{1}}=0$, then we can invert γ=0. If $\hat{\beta_{1}}$ is not equal to 0, then the other variable has an effect on the actual results, and there is a problem with the estimated equations in this paper.

**Figure 3:**
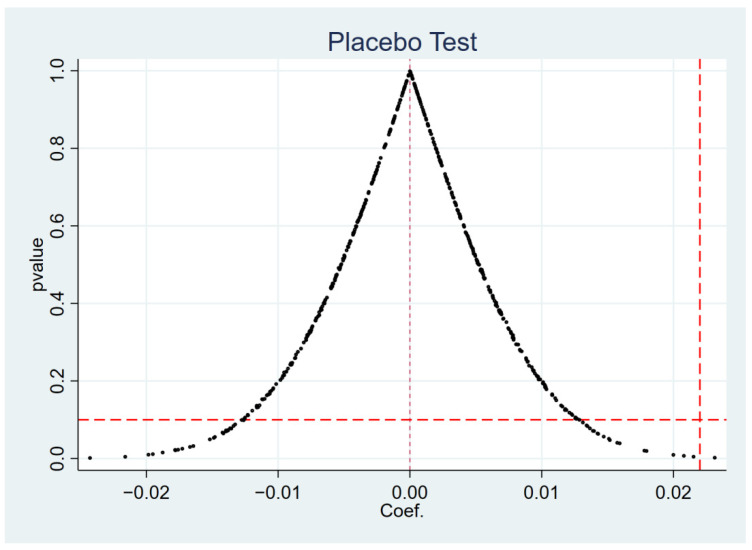
Placebo test for short-term debt financing.

**Figure 4:**
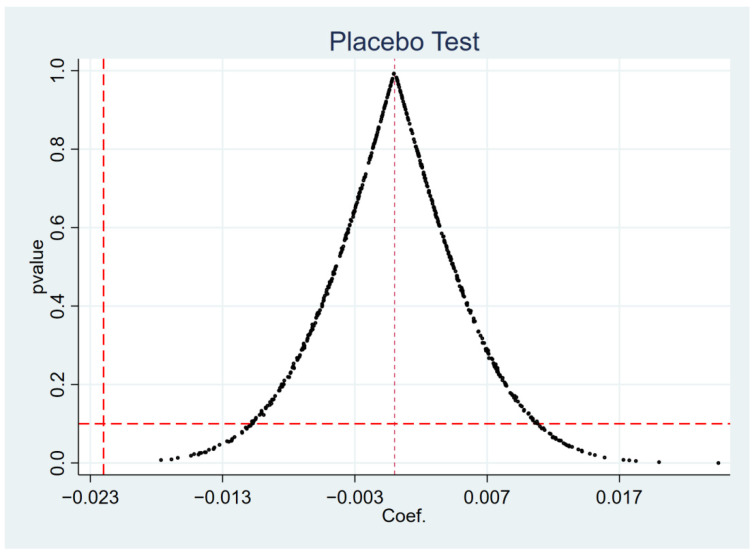
Placebo test for long-term debt financing.

Figure 3 and Figure 4 depict the *p*-value distributions for short-term debt financing and long-term debt financing. The figures show that the mean values of the estimated coefficients from the 500 simulated computer regressions are distributed around 0, and that the majority of the *p*-values are greater than 0.1. At the same time, the coefficients estimated by DID are all within the range of low-probability events in the placebo test kernel density plot. As a result, inverting γ to 0 demonstrates indirectly that the Green Credit Policy has a significant effect on the long- and short-term debt financing of heavily polluting firms, indicating that the benchmark regression results are robust.

## 6. Discussion

### 6.1. Discussion of Analysis Results

In order to explore the effect of the Green Credit Policy, this paper examines the impact of the Green Credit Policy on the short-term and long-term financing of heavily polluting enterprises from the perspective of corporate debt financing, taking the A-share listed companies from 2004 to 2020 as the sample, and uses the PSM-DID method to conduct research, drawing the following conclusions.

From the perspective of the debt financing structure, the implementation of the Green Credit Policy will have an impact on the debt financing of heavily polluting enterprises. The Green Credit Policy significantly inhibits the long-term financing of heavily polluting enterprises, which is consistent with the findings of Chai et al. [60]. After the introduction of the *Green Credit Guidelines*, there was a significant increase in short-term financing for heavily polluting enterprises, which is consistent with the findings of Zhang et al. [61]. This paper suggests that the reason for the failure of green credit to effectively control the short-term financing behavior of heavily polluting enterprises may be that, on the one hand, the regression results show that the increase in short-term financing of heavily polluting enterprises is almost synchronous with the decrease in long-term financing, suggesting that after long-term financing was blocked, firms adopted the rational financing strategy of shortening the financing period, which is consistent with the findings of Li et al. [62]. On the other hand, usually, A-share listed companies have considerable scale and high profitability, and heavily polluting enterprises are often involved in many pillar industries. Even if the capacity to finance the long-term borrowing of enterprises is blocked, enterprises can still seek more short-term financing to make up the gap in their long-term financing needs.

From the perspective of enterprise ownership, the nature of the ownership of heavily polluting enterprises affects the implementation effect of the Green Credit Policy. Compared with state-owned enterprises, the Green Credit Policy has a more significant impact on the suppression of long-term financing and the enhancement of short-term financing for non-state-owned enterprises, indicating that the Green Credit Policy does not eliminate credit discrimination against non-state-owned enterprises, which is consistent with the findings of Feng et al. [63]. State-owned enterprises account for a large proportion of the heavily polluting enterprises, and it is crucial that green credit policies work for state-owned enterprises. The asymmetry of the implementation effect of the Green Credit Policy shows that the implementation of the policy still needs to be improved.

### 6.2. Suggestions for Policy

1. Although the Green Credit Policy has a restraining effect on the long-term financing of heavily polluting enterprises, it also increases the short-term financing of heavily polluting enterprises, which weakens the implementation efficiency of the Green Credit Policy. To address this problem, the relevant state departments should clearly unify the implementation standards of the Green Credit Policy, establish clear requirements for both the long-term and the short-term financing of heavily polluting enterprises, regulate the lending behavior of financial institutions, and prevent financial institutions from enabling access to short-term credit for heavily polluting enterprises in pursuit of profits.
2. At present, the implementation effect of the Green Credit Policy is asymmetrical, and there is a problem in that the implementation effect for state-owned enterprises is not as good as that for non-state-owned enterprises. In order to solve this problem, it is necessary to clarify the supervisory responsibilities of financial departments, and strengthen supervision. The relevant state departments should construct an evaluation index system for the Green Credit Policy, and unify the evaluation standards of the policy’s implementation effect, which will help to restrain the credit behavior of banks and avoid the weakening of the policy’s effect via the ownership preferences of banks.
3. The Green Credit Policy has shown a restrictive effect on debt financing for heavily polluting enterprises, but the ultimate goal of the Green Credit Policy is to reduce environmental pollution caused by heavily polluting enterprises through green innovation, or transformation and upgrading. It often takes a long time for the transformation of heavily polluting enterprises to achieve results, and without incentives, enterprises may be trapped in a vicious cycle of continuous inadequate financing, which in turn makes it difficult for them to transform, and exacerbates pollution. In order for enterprises to respond to the Green Credit Policy and actively carry out green transformation, this paper suggests that regulators and financial institutions should give enterprises corresponding incentives according to their actual situation, such as tax relief and subsidy support for enterprises in the transformation period.

## 7. Conclusions

Compared with existing studies, the main innovations of this paper are as follows: scholars currently mainly use financing costs to measure the impact of the Green Credit Policy on the financing of heavily polluting enterprises, while some scholars also use the financing scale for this purpose, both suggesting that, overall, the Green Credit Policy places financing constraints on heavily polluting enterprises. This study introduces the short-term financing and long-term financing of enterprises as explanatory variables to explore what impact the Green Credit Policy has on the financing structure of heavily polluting enterprises, which further subdivides the research in this field, and shows that the short-term financing of heavily polluting enterprises has even increased with the implementation of the Green Credit Policy. This indicates that the Green Credit Policy is not perfectly effective in constraining short-term financing, and enriches the theoretical research in terms of the effects of credit policy implementation. In addition, this study reveals the impact of the nature of enterprise ownership on the implementation of green credit policies, with state-owned enterprises facing weaker financing constraints compared to non-state-owned enterprises. This paper provides targeted advice for the implementation of policies based on its findings, which is of great practical significance for the government as it seeks to improve the Green Credit Policy.

This study has some limitations. Firstly, only heavily polluting enterprises were chosen as the research target of this paper, but the original intention of the Green Credit Policy was not only to limit the scale of financing for heavily polluting enterprises, but also to improve the credit policy for environmentally friendly enterprises. Future research can address the impact of the Green Credit Policy on the credit of environmental protection enterprises via empirical analysis, and conduct a comparative study of the effects for heavily polluting enterprises and environmental protection enterprises, which will make the findings more comprehensive and convincing.

Secondly, although this paper confirms that the Green Credit Policy has a binding impact on the debt financing of heavily polluting enterprises, the strategic responses of enterprises when faced with credit financing constraints, the choices they will make in terms of investment, and whether green transformation can play a role in alleviating financing constraints are all issues to be addressed in future research.

## Figures and Tables

**Table 1:** Variable definitions.

Variable Type	Variable Name	Variable Symbol	Variable Interpretation
Explained variable	Short-term financing	Short-term loan	Short-term loan = short-term liabilities/total assets
	Long-term financing	Long-term loan	Long-term loan = long-term liabilities/total assets
Explanatory variable	Group virtual variable	${\mathrm{Treat}}_{i}$	The value of heavily polluting enterprises is 1, and that of non-heavily polluting enterprises is 0
	Time virtual variable	${\mathrm{Period}}_{t}$	Defined as 1 in 2012 and onwards, 0 before 2012
	Difference-in-difference variable	${\mathrm{Treat}}_{i}\times {\mathrm{Period}}_{t}$	The interaction term represents the net effect of the policy
	Nature of ownership	Ownership	[(state shares + state-owned corporate shares)/total share capital] × 100% of the value of more than 50% that state-owned enterprise valuation is 1, otherwise 0
Control variable	Age of enterprise	Age	Age of enterprise establishment
	Scale of enterprise	Size	Total assets of enterprises at year-end (logarithm taken)
	Corporate profitability	ROE	Net Profit/average stockholders’ equity
	Enterprise Development Capability (growth)	Growth	(current year main business income-last year main business income)/last year main business income
	Current ratio	C_ratio	Average current assets/average current liabilities
	Cash flow ratio	CC_ratio	NET cash flow from operating activities/current liabilities
	Cash flow interest earned ratio	CF_ratio	NET cash flow from operating activities/interest expense
	Ratio of liabilities to assets (financial risk)	LEV	Average total liabilities/average total assets of the business for the year

**Table 2:** DID model parameters.

	Before the Policy Was Implemented $\left(Period_{t}=0\right)$	After the Policy Was Implemented $\left(Period_{t}=1\right)$	Difference
Non-heavily polluting enterprise (${\mathrm{Treat}}_{i}=0$)	$Y_{\mathrm{it}}=\beta_{0}$	$Y_{\mathrm{it}}=\beta_{0}+\delta_{2}$	$\Delta Y_{1}=\delta_{2}$
Heavily polluting enterprise (${\mathrm{Treat}}_{i}=1$)	$Y_{\mathrm{it}}=\beta_{0}+\delta_{1}$	$Y_{\mathrm{it}}=\beta_{0}+\delta_{1}+\delta_{2}+\beta_{1}$	$\Delta Y_{2}=\delta_{2}+\beta_{1}$
DID			$\Delta Y=\Delta Y_{2}-\Delta Y_{1}=\beta_{1}$

**Table 3:** Matching effect test.

	Unmatched	Mean			%Reduct	*t*-Test		V(T)/
Variable	Matched	Treated	Control	%bias	|bias|	*t*	*p* > *t*	V(C)
age	U	26.54	26.59	−1.400		−0.650	0.517	0.88 *
	M	26.543	26.518	0.7	50.200	0.310	0.758	0.89 *
C_ratio	U	1.0282	1.2526	−38.000		−17.780	0.000	1.060
	M	1.0285	1.0473	−3.200	91.600	−1.480	0.139	1.30 *
CC_ratio	U	0.173	0.092	39.900		18.750	0.000	1.32 *
	M	0.172	0.169	1.8	95.500	0.750	0.454	1.000
CF_ratio	U	4.621	3.968	6.7		3.110	0.002	0.66 *
	M	4.620	4.927	−3.200	53.000	−1.540	0.123	0.92 *
lev	U	0.571	0.584	−7.800		−3.640	0.000	0.990
	M	0.571	0.555	9.699	−24.700	4.400	0.000	1.07 *
roe	U	0.044	0.046	−1.200		−0.560	0.575	1.17 *
	M	0.044	0.039	3.3	−173.200	1.430	0.153	1.10 *
growth	U	0.172	0.191	−4.000		−1.840	0.066	0.78 *
	M	0.172	0.163	1.7	56.800	0.800	0.425	0.93 *
size	U	22.531	22.322	15.700		7.340	0.000	1.020
	M	22.529	22.403	9.5	39.600	4.160	0.000	0.960

* if variance ratio outside [0.94; 1.06] for U and [0.94; 1.06] for M.

**Table 4:** Descriptive statistics for each variable.

	(1)	(2)	(3)	(4)	(5)
Variables	N	Mean	SD	Min	Max
ownership	8797	0.615	0.487	0	1
Short-term loan	8797	0.174	0.111	0	0.534
Long-term loan	8797	0.0844	0.102	0	0.465
age	8797	26.57	3.631	20	36
C_ratio	8797	1.150	0.594	0.184	4.498
CC_ratio	8797	0.128	0.205	−0.402	1.024
CF_ratio	8797	4.266	9.794	−24.67	53.49
lev	8797	0.578	0.163	0.160	1.100
roe	8797	0.0454	0.159	−0.859	0.388
growth	8797	0.183	0.488	−0.579	3.478
size	8797	22.41	1.335	19.43	25.98

**Table 5:** DID test of the impact of the Green Credit Policy on short-term and long-term financing.

	(1)	(2)	(3)	(4)
Variables	Short-Term Loan	Short-Term Loan	Long-Term Loan	Long-Term Loan
did	0.023 ***	0.022 ***	−0.029 ***	−0.023 ***
	(4.95)	(5.41)	(−6.66)	(−6.09)
treat	0.002	0.006 *	0.050 ***	0.032 ***
	(0.45)	(1.94)	(15.23)	(11.56)
period	−0.050 ***	−0.029 ***	0.009 ***	−0.019 ***
	(−16.05)	(−10.03)	(3.59)	(−7.58)
age		0.000		0.000
		(0.85)		(1.09)
C_ratio		−0.037 ***		0.017 ***
		(−18.61)		(8.42)
CC_ratio		−0.071 ***		0.218 ***
		(−10.31)		(26.97)
CF_ratio		−0.001 ***		−0.003 ***
		(−5.89)		(−19.91)
lev		0.156 ***		0.207 ***
		(17.67)		(25.46)
roe		−0.042 ***		0.002
		(−4.41)		(0.33)
growth		−0.010 ***		0.003
		(−4.16)		(1.56)
size		−0.019 ***		0.022 ***
		(−19.40)		(26.82)
Constant	0.194 ***	0.561 ***	0.064 ***	−0.566 ***
	(82.52)	(25.70)	(35.18)	(−30.46)
Observations	8797	8797	8797	8797
R-squared	0.038	0.271	0.034	0.310
F test	0	0	0	0
r2_a	0.0379	0.270	0.0339	0.309
F	121.3	257.9	93.23	245.6

Robust *t*-statistics in parentheses. *** *p* < 0.01, * *p* < 0.1.

**Table 6:** Test of green credit on enterprise financing under the control of enterprise ownership.

	State-Owned	Non-State-Owned	State-Owned	Non-State-Owned
Variables	Short-Term Loan	Short-Term Loan	Long-Term Loan	Long-Term Loan
did	0.022 ***	0.026 ***	−0.021 ***	−0.026 ***
	(4.04)	(4.05)	(−4.40)	(−4.50)
treat	0.010 **	−0.002	0.028 ***	0.040 ***
	(2.47)	(−0.40)	(7.64)	(9.41)
period	−0.020 ***	−0.047 ***	−0.024 ***	−0.010 ***
	(−5.22)	(−10.09)	(−7.23)	(−2.66)
age	0.001 **	−0.001	0.000	0.000
	(2.10)	(−1.14)	(0.40)	(1.30)
C_ratio	−0.034 ***	−0.043 ***	0.017 ***	0.018 ***
	(−12.49)	(−14.46)	(6.34)	(5.82)
CC_ratio	−0.060 ***	−0.081 ***	0.214 ***	0.220 ***
	(−6.81)	(−7.37)	(21.40)	(16.65)
CF_ratio	−0.001 ***	−0.000	−0.003 ***	−0.003 ***
	(−6.62)	(−1.48)	(−14.97)	(−13.10)
lev	0.176 ***	0.125 ***	0.200 ***	0.218 ***
	(15.39)	(8.93)	(19.02)	(17.06)
roe	−0.038 ***	−0.051 ***	−0.012	0.026 **
	(−2.97)	(−3.52)	(−1.24)	(2.29)
growth	−0.010 ***	−0.008 **	0.005 *	0.001
	(−3.29)	(−2.49)	(1.74)	(0.44)
size	−0.021 ***	−0.014 ***	0.024 ***	0.019 ***
	(−17.82)	(−8.79)	(22.63)	(14.66)
Constant	0.584 ***	0.522 ***	−0.600 ***	−0.515 ***
	(20.54)	(15.09)	(−24.58)	(−17.91)
Observations	5408	3389	5408	3389
R-squared	0.264	0.293	0.301	0.330
F test	0	0	0	0
r2_a	0.263	0.291	0.300	0.327
F	145.1	133.6	154.4	92.22

Robust *t*-statistics in parentheses. *** *p* < 0.01, ** *p* < 0.05, * *p* < 0.1.

**Table 7:** Robustness test regression results after the replacement of matching methods.

	1:4 Neighborhood Match		Caliper Match	
Variables	Short-Term Loan	Long-Term Loan	Short-Term Loan	Long-Term Loan
did	0.026 ***	−0.021 ***	0.023 ***	−0.023 ***
	(5.98)	(−5.48)	(5.49)	(−6.25)
treat	0.005	0.029 ***	0.006 *	0.033 ***
	(1.52)	(9.97)	(1.88)	(11.69)
period	−0.033 ***	−0.017 ***	−0.029 ***	−0.019 ***
	(−10.40)	(−6.28)	(−10.03)	(−7.57)
age	0.000	0.000	0.000	0.000
	(1.12)	(0.77)	(0.88)	(1.07)
C_ratio	−0.036 ***	0.017 ***	−0.038 ***	0.018 ***
	(−17.22)	(7.51)	(−18.91)	(8.63)
CC_ratio	−0.063 ***	0.210 ***	−0.068 ***	0.213 ***
	(−8.48)	(24.33)	(−9.85)	(26.29)
CF_ratio	−0.001 ***	−0.003 ***	−0.001 ***	−0.003 ***
	(−5.87)	(−17.90)	(−6.13)	(−19.61)
lev	0.159 ***	0.198 ***	0.157 ***	0.207 ***
	(16.65)	(22.70)	(17.75)	(25.32)
roe	−0.046 ***	0.004	−0.042 ***	0.002
	(−4.61)	(0.55)	(−4.37)	(0.22)
growth	−0.009 ***	0.003	−0.010 ***	0.003
	(−3.43)	(1.38)	(−4.16)	(1.49)
size	−0.018 ***	0.022 ***	−0.019 ***	0.022 ***
	(−17.71)	(24.97)	(−19.45)	(26.87)
Constant	0.553 ***	−0.556 ***	0.562 ***	−0.567 ***
	(23.16)	(−27.98)	(25.74)	(−30.44)
Observations	7629	7629	8796	8796
R-squared	0.274	0.287	0.271	0.303
F test	0	0	0	0
r2_a	0.273	0.286	0.270	0.303
F	228.2	196.7	258.9	240.5

Robust *t*-statistics in parentheses. *** *p* < 0.01, * *p* < 0.1.

## Data Availability

Not applicable.
